# Improving the use of health data for health system strengthening

**DOI:** 10.3402/gha.v6i0.20001

**Published:** 2013-02-13

**Authors:** Tara Nutley, Heidi W. Reynolds

**Affiliations:** 1 MEASURE Evaluation, Futures Group, Chapel Hill, NC, USA; 2 MEASURE Evaluation, Carolina Population Center, University of North Carolina, Chapel Hill, NC, USA

**Keywords:** data-informed decision making, logic model, guidance, health information systems, health systems strengthening, data use

## Abstract

**Background:**

Good quality and timely data from health information systems are the foundation of all health systems. However, too often data sit in reports, on shelves or in databases and are not sufficiently utilised in policy and program development, improvement, strategic planning and advocacy. Without specific interventions aimed at improving the use of data produced by information systems, health systems will never fully be able to meet the needs of the populations they serve.

**Objective:**

To employ a logic model to describe a pathway of how specific activities and interventions can strengthen the use of health data in decision making to ultimately strengthen the health system.

**Design:**

A logic model was developed to provide a practical strategy for developing, monitoring and evaluating interventions to strengthen the use of data in decision making. The model draws on the collective strengths and similarities of previous work and adds to those previous works by making specific recommendations about interventions and activities that are most proximate to affect the use of data in decision making. The model provides an organizing framework for how interventions and activities work to strengthen the systematic demand, synthesis, review, and use of data.

**Results:**

The logic model and guidance are presented to facilitate its widespread use and to enable improved data-informed decision making in program review and planning, advocacy, policy development. Real world examples from the literature support the feasible application of the activities outlined in the model.

**Conclusions:**

The logic model provides specific and comprehensive guidance to improve data demand and use. It can be used to design, monitor and evaluate interventions, and to improve demand for, and use of, data in decision making. As more interventions are implemented to improve use of health data, those efforts need to be evaluated.

Strengthening of health systems has become a top priority of many global and national health agendas as a way to improve health outcomes. With the global health context becoming increasingly complex, national health systems are beginning to move away from a focus on disease-specific health responses to comprehensive strengthening of health systems. The global community agrees that without a systems approach, health outcomes will not further improve and health-related development goals such as the United Nation's Millennium Development Goals (MDGs) for 2015 will not be met (1, 2).

The World Health Organization (WHO)'s framework for health systems strengthening identifies six attributes of a health system (3). The attributes, or building blocks, include a health workforce; health services; health financing; governance and leadership; medical products, vaccines, and technologies; and health information. While each building block of the WHO framework is important to improving health systems and ultimately health outcomes, quality and timely data from health information systems (HIS)^1^ are the foundation of the overall system and inform decision making in each of the other five building blocks in the health system (4). For example, for a workforce to be trained and deployed in adequate numbers to deliver quality services, information about disease burden, the geographic distribution of target groups, and available infrastructure and commodities is necessary. Health systems require quality data from HIS to plan for and ensure that the workforce is fully funded and equipped with the necessary commodities, infrastructure, resources, and policies to deliver services.

Quality health data are, in and of themselves, prerequisites to improving each of the other five building blocks of the health systems (5). Because programs often fall short of efficient use of data to inform decisions, international commitments have been made to strengthen the quality, relevance, and comprehensiveness of data to ultimately improve data use and data-informed decision making (6). Positive experiences using data in turn contribute to a demand for additional data and a continued commitment to improving the quality of data and continued data use. The relationship of improved information, demand for data, and continued data use creates a cycle that leads to improved health programs and policies (7).

The ‘use’ of data is the analysis, synthesis, interpretation, and review of data as part of a decision-making processes, regardless of the source of data. We focus on the demand for and use of data as captured in various data sources such as surveys and other research efforts, civil registers, and routine health information systems (RHIS). ‘Data-informed decision making’, then, refers to the proactive and interactive processes that consider data during program monitoring, review, planning, and improvement; advocacy; and policy development and review (7).

The objective of this article is to employ a logic model to describe a pathway of how specific activities and interventions can strengthen the use of health data in decision making in order ultimately to fortify the other building blocks of the health system. The article builds on previous work in the field by making specific recommendations about interventions that are most proximate to affect the use of data in decision making. The logic model with activities and examples of their implementation provide a practical strategy for developing, monitoring, and evaluating interventions to strengthen the use of data in decision making.

## Methods

### The underutilization of data in decision making

Too often data sit in reports, on shelves, or in databases and are not sufficiently used in program development and improvement, policy development, strategic planning, or advocacy. Part of the reason for the breakdown in the process is that HIS are inherently complex, and the outputs of HIS (quality data) are not proximately related to improved service delivery (8). The output of improving the health workforce, for example, is directly related to improvements in service quality and coverage, while the output of improved information systems is higher quality and timely data. The complexity of how organizations are contributing to and using HIS (9), of decision-making processes, of the flow of information, and of the time lag between the availability of data and use of data and the eventual changes in services and health outcomes all contribute to a breakdown in the causal pathway and an underutilization of data in decision making (8, 10). The existence of quality data is insufficient to ensure use (4) because data use has not been adequately integrated into decision-making processes and the information needs of decision makers are often not adequately represented in data collection efforts (11). Without specific policies and interventions aimed at improving the use of data produced by information systems; health systems will never fully be able to meet the needs of the populations they serve. To date, clear guidance on how to comprehensively improve data-informed decision making is lacking.

### Logic model

**Fig. 1:**
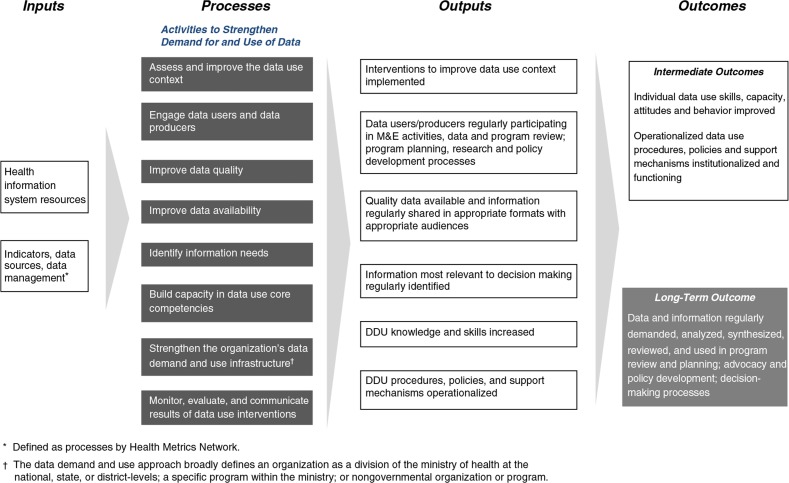
Logic model for strengthening the use of health data in decision making.

The objective of this article is to use a logic model (Fig. 1) to describe specific activities and interventions and describe how they improve the use of health data. A logic model describes the main components of an intervention and how they are intended to work together to reach measurable objectives. Use of a logic model allows for critical assessment of program impact pathway theory and assumptions; appropriateness and completeness of activities (process); and indicators of outputs (direct products of program activities), outcomes (specific changes in program participants’ behavior, knowledge, skills, and level of functioning), and impacts (the fundamental intended or unintended change occurring in organizations, communities or systems as a result of program activities) (12).

The logic model presented in this article maps out how the intervention inputs and activities are expected to influence the outputs and eventual outcome of regular data use in program review, planning, advocacy, policy development and other decision making processes. It can help to specify the theoretical assumptions under which the intervention is intended to influence outcomes. It can help identify gaps in programming, and as data are gathered, it can help identify areas for programmatic strengthening. It can be used to decide which program areas need to be studied systematically to gain information about whether the program assumptions are correct. The logic model in Fig. 1 acts as a roadmap for how a set of eight interventions can affect the regular use of data in decision making.

The logic model was developed by a working group of monitoring and evaluation (M&E), research and data-use experts as part of the MEASURE Evaluation Project. The group met on multiple occasions to define and refine the model. The logic model is currently being applied and tested as the organizing construct for data use activities in the MEASURE Evaluation Project. The activities that are included in the logic model as interventions to strengthen the demand for and use of data include:

1. assess and improve the data use context;
2. engage data users and data producers;
3. improve data quality;
4. improve data availability;
5. identify information needs;
6. build capacity in data use core competencies;
7. strengthen the organization's data demand and use infrastructure; and
8. monitor, evaluate, and communicate results of data use interventions.

The activities to strengthen the demand for and use of data are built upon a foundation of inputs. For the purposes of the logic model, these inputs are informed by the inputs and processes of health information systems (HIS) defined by the Health Metrics Network (13) because efforts to improve the demand for and use of information will only be successful if these efforts are implemented within a HIS that is functioning or in the process of being strengthened. Thus, the inputs for our logic model include legislative, regulatory and planning frameworks; resources including personnel, financing, logistics support, information and communications technology; and indicators, data sources, and data management.

The activities to improve the use of data are informed by previous work; building primarily on four major works in the field:

1. Aqil and colleagues (14) developed the Performance of Routine Information System Management (PRISM) framework to improve routine health information systems (RHIS) and data use. The framework is innovative in that it puts emphasis on RHIS performance and the three interrelated determinants of that performance: technical, behavioral, and organizational determinants. The technical refers to systems such as data collection processes, systems, and methods. The behavioral refers to the behaviors of data users and how data are used for problem solving and program improvement. The organizational refers to the structure and processes of the organizations that use the resulting information. PRISM emphasizes that specific technical, behavioral, and organizational activities need to be implemented to improve demand for, analysis, review, and use of routine health data in decision making.
2. The Health Metrics Network (13, 15) approaches the strengthening of the entire HIS, which among many other things, includes improving the use of data in decision making. The network is unique in that it is the first global health partnership that focuses on core requirements of HIS. The framework lays out a standard for guiding the collection, reporting and use of health information by all developing countries and global agencies.
3. Lomas in his foundational article (11) promotes improved communication between those that generate research data and those that use research data in decision making. He states that an overemphasis on trying to change the behavior of health practitioners to use data is resulting in a failure to consider other stakeholders in the research and data use processes. He promotes a multidisciplinary approach to improve understanding of the environment in which research is generated and the policy process and context that puts it into practice.
4. Patton promotes strategies for increasing the use of data in decision making that are generated from evaluation research. In his 2008 textbook (16), he comprehensively provides both theoretical and practical guidance for how to conduct program evaluations that will produce results that are useful to improving health programs.

Each author addresses data use from their own ‘data perspective’. The Health Metrics Network (HMN) has the broadest perspective and considers data captured in various data sources. Common data sources in HIS include data from population-based surveys and civil registers and from the operations of institutions that deliver health services (most commonly health facilities), health data generated through administrative, management, and logistical process of those institutions that support the delivery of health services (e.g. labor, finances, and commodities). Data sources from sectors that also affect health, for example, food and agriculture, and those organizations that report select health outcomes (e.g. police) are also rich sources that contribute to HIS (13). PRISM addresses data use from routine health information systems which include any data collection conducted regularly with an interval of less than 1 year in health facilities and their extension in the community (14). Lomas considers use of data that are generated by specific research endeavors (11, 17). Similarly, Patton addresses data use in the context of program evaluation (16). The data sources addressed by Lomas and Patton are not routinely collected as RHIS data are, but compliment routinely collected data when considered together. Both routinely and non-routinely collected data make up the data sources in a HIS.

While all of these authors have substantially contributed to the field of improving data use in decision making, it is challenging for the end practitioner to pull out the ‘how to’ when each author approaches the topic from different data perspectives and different levels of detail. This article builds on these previous works by drawing on their collective strengths and similarities and proposes specific interventions that are most proximate to affect the use of data in decision making. Table 1 lists the eight interventions proposed in this article and summarizes whether or not the authors listed above promoted similar approaches. The checks (‘√’) indicate that the authors considered the activity as important to facilitating the use of information and address the activity in their work. The ‘X's indicate that there was no mention of the activity or that it was only cursorily addressed. There are not instances where the authors’ work contradicted the proposed intervention.

**Table 1:** Influential thinker's contributions to identifying activities to strengthen use of data in decision making

Activities to strengthen demand for and use of data	HMN (4, 15)	PRISM (13, 14)	Lomas (12)	Patton (16)
	
	Addresses: Health Information Systems	Addresses: Routine Health Information Systems	Addresses: Research	Assesses: Evaluation
Assess and improve the data use context	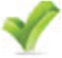	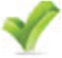	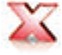 a	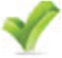 b
Engage data users and data producers	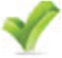	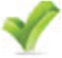	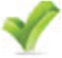	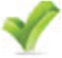
Improve data quality	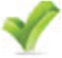	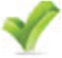	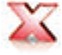 c	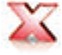 c
Improve data availability (access, synthesis, communication)	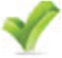	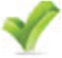	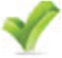 d	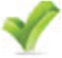 e
Identify information needs	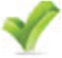	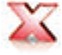 f	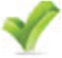	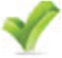
Build capacity in data use core competencies^g^	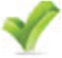	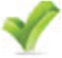	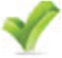	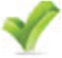
Strengthen organizations data demand and use infrastructure	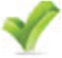	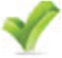	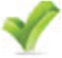	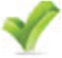 h
Monitor, evaluate and communicate results of data use interventions	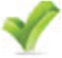	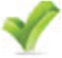	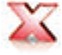	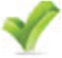

a Does not mention assessing data use context or activities that facilitate data use.

b Recommends conducting a readiness assessment (for implementing a utilization focused evaluation) and provides checklists and other tools to assist in the process. Does not provide an assessment tool.

c Data quality is not an issue in primary data collection as it is in routine information sources; therefore, it is not addressed by the authors.

d Does not mention of the importance of having access to the data generated by the study beyond synthesized information and key findings.

e Recommends involving intended users in data analysis and interpretation but does not discuss having access to data set after study completion.

f Includes one question that asks: ‘Do you think that the information system design provides a comprehensive picture of health system performance’.

g No common set of data use core competencies, but all authors stress the importance of building skills. Some authors list competencies.

h Recommends assessing and improving a program/organization's readiness to do utilization-focused evaluations as well as ways to overcome barriers to using evaluation results. No specific guidance for how to do this. All recommendations are specific to evaluations.

The logic model (Fig. 1) provides a framework for implementing, monitoring and evaluating the interventions listed in Table 1 to achieve the regular demand, analysis, synthesis, review, and use of data in program review and planning; advocacy and policy development and other decision-making processes. By linking outcomes to specific activities in the logic model, a clear causal pathway is built for how investments in activities to improve the demand for and use of data result in improved data-informed decision making. Ultimately, improved decision making will strengthen the health system and improve health outcomes (5). The following section defines the activities to strengthen data use in more detail and provides examples of their implementation so that outputs and outcomes can be translated into indicators to evaluate the effect of activities to strengthen data demand and use.

## Activities and examples to strengthen the demand for and use of data

This article recommends specific interventions to strengthen data demand and use and describes how the interventions influence outputs and outcomes in the logic model (Fig. 1). This section cites specific examples for each intervention to increase the likelihood that future interventions will be designed, implemented, and tested. The examples presented also highlight how each intervention leads to various outputs and outcomes in the logic model (Fig. 1).

### Assess and improve the data use context

Assessment of the organizational, technical, and behavioral factors that affect decision making is necessary to diagnose where to intervene with activities to improve demand for and use of data. Most assessments of health system functioning, with the exception of PRISM tools, assess information dissemination and use (14, 15, 18), but fall short of in-depth analyses of the organizational and behavioral factors that affect the role of data and information in decision making. This information is needed to comprehensively improve data-informed decision making.

Examples come from the application of the PRISM and Health Metrics Network framework and tools in Mexico, Brazil, Honduras, Paraguay, Dominican Republic, Peru, and Ecuador from 2005 to 2012 (19). Results of these assessments revealed that countries struggled with adequacy of resources, data sources, information products, and dissemination and use of data. In response to these findings, countries developed and implemented their own strategies to strengthen HIS. Specifically, countries have sought to secure stakeholder buy-in and funding to implement their health strategic plans; designed and developed databases at the sub-national and health facility levels; made databases available to the public; and developed capacity building and training programs that addressed data and information, such as a master's degree program in public health with a focus on biostatistics and HIS in Mexico.

Another data use outcome of the assessment process was the creation of the Latin American Network to Strengthen HIS (RELACSIS, www.relacsis.org ). RELACSIS serves as a platform for countries to share information and learn from others’ experiences in strengthening HIS and disseminating and using information. RELACSIS was launched in April 2010 and country teams were formed. The most recent focus has been on capacity building of health personnel on improving the quality and analysis of morbidity and mortality data through the use of the International Classification of Diseases, version 10.

It is also possible to apply assessment tools like PRISM to identify areas for HIS improvement and then to use the tools again to assess changes over time. In Pakistan, the PRISM framework and tools were used at baseline to inform a package of interventions to reform the HIS. The tools were used after a pilot test period to assess changes over time (20). The PRISM framework instruments have been found to be reliable and valid, suggesting they can be used for monitoring changes in data quality, use of information, processes and competencies, and to promote of a culture of information (21).

### Engage data users and data producers

Typically it is people in various job functions and at different levels of the health system who address efforts to collect, analyze, synthesize, interpret, and use data in decision making. The lack of interaction between individuals who design and manage research and information systems – the data producers – and professionals who use data in program improvement and development – the data users – contributes to the breakdown in the decision-making cycle (11, 17). When data users and data producers work together, they become more aware of the data collection processes and methods, the available data sources, and the quality of those data. They have the opportunity to address barriers to data use and improve the sharing of data resources. They can also discuss concerns and seek clarification about the data collection process (16), and identify key programmatic questions and link these questions to the data available in their settings. They can jointly analyze and interpret data to answer programmatic questions. By understanding who your data users and producers are and linking them to each other's work, ownership of data is clarified, the information cycle is strengthened, data-informed decisions are made, and the value of data in relation to program improvement becomes clear (11, 16, 17, 22–24).

An example of data users and producers working together comes from Madagascar, where policy makers at the Ministry of Health and Family Planning (MOHFP) worked with researchers to link key program questions with the available data and jointly analyze and interpret data. The MOHFP was interested in the strategy of allowing community based workers to distribute the injectable contraceptive method depot medroxyprogesterone acetate (DMPA), but they wanted research evidence that quality DMPA services could be provided by community workers and that this method of delivery was acceptable. At the beginning of the study to assess safety of the strategy, different data users were identified, such as providers and policy makers, and they were included in the development of study questions to ensure that the research addressed their questions and concerns. These stakeholders were invited to join a study advisory committee, and there were regular communications during the study with this group. Stakeholders were given meaningful roles in the study during data collection to help increase their understanding of the research process. During data analysis, stakeholders were involved in data interpretation and gave rich context to the results, and development of recommendations was led by stakeholders. This process to link data users and data producers was considered successful in that the intervention allowing community based workers to distribute DMPA was eventually adopted by the MOHFP and was scaled up (25).

### Improve data quality

For consistent data use to occur, data need to be of high quality so that data users are confident that the data they are consulting are accurate, complete, and timely. Without quality data, demand for data drops, data-informed decision making does not occur, and program efficiency and effectiveness will suffer (7, 26, 27). Data quality protocols need to be developed, communicated, and implemented, as well as training and retraining of health professionals on data quality techniques and approaches.

An example of activities to improve data quality comes from the field of routine immunization services. Bosch-Capblanch and colleagues reviewed 41 countries’ data quality performance from 2001 to 2005 (28). Six of 41 countries had two rounds of data quality assessments (DQA) 2–3 years apart because they failed to meet the accuracy targets in the first round and had to produce data quality improvement plans. During the second rounds of DQA, it was observed that the accuracy and quality of the reporting system improved, there was an increase in the availability of guidelines for electronic data management, and demonstrated better use at the district level of immunization performance monitoring tools (e.g. tables and charts showing coverage).

### Improve data availability

Data availability, defined by the authors as data synthesis, data communication, and access to data, all need to be improved to support the use of the information in decision making (14). To ensure that data are understood by potential users, data synthesis and communication need to be targeted and take into account users’ roles and information needs, the appropriate level of detail and complexity of the information being presented, and users’ intensity of interest in the topic (29). Well-designed information systems should include the information technology infrastructure, policies, and report templates to support the targeted communication of synthesized data through dissemination and feedback techniques. Moreover, data users need to be able to access and share data easily outside of the regular dissemination process.

Examples include electronic information systems and medical records that work to make data more available by increasing access and expanding the uses of those data. Integrated HIS can transform fragmented unidirectional indicator reporting systems to shared data that can be analyzed to improve service delivery and continuum of care for clients (30). An evaluation of a computerization of the health management information system in a community in India documented that the implementation of the system increased data quality, improved data storage and management, resulted in a better tool to support monitoring and supervision activities, saved time, and improved service delivery, aided in report generation and better monitoring of indicators (31). Moreover, while the implementation of the computerized health management information system strengthened data storage, service delivery, program monitoring and reporting, it expanded the uses of the data to research as data are available for an entire population and over time.

Another example of improving data availability is from work in Uganda where the Ministry of Health (MOH) wanted to scale up the practice of community based distribution (CBD) of DMPA to the public sector. The MOH provided advocacy literature to all districts summarizing the results of research from multiple settings of the safety and efficacy of CBD provision of DMPA. They also provided job aids and a list of steps to initiate the practice within existing CBD program, and they offered to support local health officials to replicate the practice in their district (32). Seven districts opted to receive support implementing the program. Following the intervention and technical support, women's access to the method increased.

### Identify information needs

Information systems are developed to meet the needs of multiple data users throughout a health system. Because of the many types of data users that access information systems and their diverse needs, the resulting data may not necessarily respond to the specific information needs of all potential data users (29). Moreover, the vast amount of information may be overwhelming to the potential users who are ill equipped to navigate the data resources available to them. To facilitate data use, a focus needs to be placed on what stakeholders need to know to effectively run health programs, on the practical questions data users have about their programs, and the upcoming decisions that they have to make. With this knowledge they are able to focus on collecting information that is directly linked to decision making. (16, 24, 33).

The government of Afghanistan applied this thinking to the management of their Basic Package of Health Services (BPHS) in 2004 (34, 35). After decades of conflict, the health system, including the routine system that collected information on health services, was in ruins. To monitor the implementation of the BPHS, the Ministry of Public Health (MOPH) chose to initiate household surveys and annual surveys of health facilities, and to use a balanced scorecard (BSC) to benchmark progress. The scorecard honed in on the MOPH's most relevant information needs through the selection of 29 indicators out of the 340 potential indicators to include in the scorecard. The indicators selected were chosen because they were easily understood, robust, and represented the most important aspects of the program. The BSC was designed via a participatory series of workshops and discussions with the MOPH, non-governmental organizations, and other development partners active in the health sector, including health workers and managers. In the eyes of the MOPH, the BSC demonstrated open and rational decision-making.

The MOH used the BSC to identify eight priority health areas in need of improvement, based on the unsatisfactory level of performance for each indicator and its importance to the MOPH's strategy to improve health. For each indicator, upper and lower benchmarks were set. The BSC gives each province, and the country as a whole, an indication of how they are performing in delivering the BPHS, even though it has some limitations. Using the BSC to assess performance of the health sector over time, target program improvement efforts and identify best practices in priority areas will enable the MOPH and stakeholders to make evidence-informed decisions that will advance Afghanistan's health goals (34, 35).

### Build capacity in data use core competencies

To improve sustainable demand for and use of data in decision making, individual capacity in core competencies to demand and use data must exist at all levels of the health system. Competencies include skills in data analysis, interpretation, synthesis, and presentation, and the development of data-informed programmatic recommendations. For data producers, these competencies should be built as part of standard monitoring and evaluation (M&E) training or basic research training, but often training programs have a short-term perspective (1–4 weeks) with limited follow up. Skills are not fully developed and newly trained professionals are underequipped to apply their new skills in the work setting (36). M&E and research capacity building programs also tend to place a greater focus on developing and managing M&E systems and research studies with little or no pedagogic emphasis on using those data in decision making.

Moreover, the target audience for M&E and research training is the data producer not the data user. Data users often struggle with an underdeveloped ability to understand analyses and interpret them in the programmatic context. This population also needs to be targeted with training in how to analyze, critically review, and interpret data and understand what data they need and when they can demand data. For data-informed decision making to become normative and sustained, funding will be needed to implement and sustain the interventions outlined in this article. Training in leadership and advocacy skills is critical to equip managers to leverage the funding and buy-in needed to implement and sustain interventions to improve demand for and use of data.

An example of how building skills in how to analyze, interpret, synthesize, and present data comes from South Africa. The University of Witwatersrand is located near the Agincourt Demographic Surveillance Site, a site that produces data on demographic events from over 82,000 people in 26 villages in the sub-district of Agincourt, South Africa. To build the capacity of students at the University of Witwatersrand, their training programs were given access to the Agincourt surveillance data. Courses were tailored to build students’ skills in the data management, analysis, and research skills of longitudinal data (37). Making the data available to students also resulted in solutions that actually made the data more available to the public in general (an example that is relevant for the ‘improving data availability’ section above). A data extraction program was used to create an anonymized 10% sample database along with the documentation so that potential users would be able to gain a better idea of the study and data set contents, and improve their requests and proposals for using the data. This experience is informing other training models.

### Strengthen the organization's data demand and use infrastructure

All organizations are made up of people and the effectiveness of an organization is directly linked to the performance of its employees (38); most organizations are governed by rules, processes, values, and systems that have the ability to support or hinder an individual's ability to use data in decision making (14, 39). For example, an organization that has structures and processes for improving the interaction of data users and producers, providing clear guidelines for data quality processes, and defining roles and responsibilities related to using data will strengthen other interventions put in place (such as those outlined in this article) to improve data-informed decision making. An organization that has a guiding strategy and mission that clearly supports data-informed decision making will be better positioned to support data-informed decision making. Policies and standard operating procedures that govern how work is accomplished should clearly state the role and value of data in organizational functioning. By addressing organizational systems, such as those just mentioned, potential barriers to data use can be overcome and data-informed decision making can be improved and sustained.

A theoretical model of organizational influences on nurses’ research utilization was tested in Alberta, Canada (no studies were found from developing countries) and results point to how a supportive and positive organization and context influence research utilization (40). Responsive administration (such as supporting nurses through providing resources and promoting autonomy) and relational capital (such as collaborative relationships between clinicians) were organizational characteristics that influenced nurses’ research utilization. These factors worked to increase research utilization by increasing staff development and reducing emotional exhaustion. Moreover, for nurses working in contexts with a positive culture, good leadership and feedback on their work increased research utilization and resulted in fewer adverse events in the workplace.

### Monitor, evaluate, and communicate results of data use interventions

In order for stakeholders and decision makers to use data in decision making, they need to place value on data (41). This value can be built through a positive experience using information to support a decision, through training or through exposure to positive messages about the benefits of using data in the decision-making process (7). The higher the value data users put on data-informed decision making, the more likely they are to use data. Regular use of data in decision making generates demand for quality data and the reinforcement of data-informed decision-making processes. Through the evaluation of data demand and use interventions and the communication of data demand and use successes, the knowledge base is built for substantiating investments in interventions to strengthen data demand and use.

The reaching every district (RED) approach illustrates the value of evaluating data use interventions (42). RED has five components intended to improve capacity at the district and health facility levels to address obstacles and improve immunization services. One of the five components is ‘monitoring data for action’ and stresses the use of data to analyze program status and modify activity plans. Other components include planning and management, supervision, outreach, and community linkages. A five-country evaluation of the RED approach found that health facilities and offices had catchment area maps, health workers were able to describe the population and challenges to reaching them with immunizations, there were defaulter tracking systems used to reach children, wall monitoring charts were displayed, and district level meetings were held to review program status (42). One of the major challenges across all countries was having accurate denominators that affect health worker's ability to target interventions. The evaluation suggested that the RED approach was implemented and the immunization program strengthened at the same time that immunization coverage increased, although it is not possible to know what component was the most effective nor to what extent the RED approached caused the changes in immunization rates.

## Summary and conclusions

This article stresses the central role that HIS play in strengthening health systems while at the same time underscores the insufficient reliance on data in health decision making. The lack of demand for and use of data limits the health system's ability to respond to priority needs throughout its many levels. The failure to consider empirical evidence regularly before making program and policy decisions is due primarily to the complex causal pathway between data collection, use of data, and improvement in health outcomes. Furthermore, specific and comprehensive guidance to improve data demand and use is lacking. This article fills this gap by providing specific recommendations for how to improve data-informed decision making by suggesting domains of activities and by providing examples of how they have been applied in other contexts. The eight activity areas listed in the logic model provide a comprehensive roadmap for how to design, monitor, and evaluate interventions to improve the demand for and use of data in decision making.

More experience is needed applying the logic model in different contexts. The factors influencing demand for and use of data are dependent on the local context and specific needs. The logic model is not meant to imply that all activities and equal intensity of implementation are necessary to improve data use. Rather, depending on the context, all of the activity areas discussed in this article may not need to be implemented as part of an intervention to improve the demand for and use of data. Similarly, upon further application, additional activity areas not listed here may be identified. The relative importance of each activity area is unknown, as is the level of intensity of each activity area. Nonetheless, this logic model contributes to the literature on comprehensive approaches to improving the use of data in decision making.

As more interventions are implemented with the aim to improve use of health data, those efforts need to be evaluated. There is limited understanding in general whether interventions implemented based on assessment findings are actually implemented as planned, and whether the interventions resulted in anticipated changes in HIS in general and data demand and use specifically. More systematic use of PRISM over time to assess and inform changes is needed, but other data collection methods are also relevant.

## Footnotes

1 Health Information Systems (HIS) are comprised of health information system resources, indicators, data sources, data management, information products, dissemination and use (Health Metrics Network. Framework and Standards for Country Health Information Systems. Health Metrics Network: World Health Organization, 2008).
